# The state of the art on treatment of Crohn’s disease

**DOI:** 10.1007/s00535-018-1479-6

**Published:** 2018-07-06

**Authors:** Hai Yun Shi, Siew Chien Ng

**Affiliations:** 1 Department of Gastroenterology, Beijing Friendship Hospital, Capital Medical University, National Clinical Research Center for Digestive Diseases, Beijing, China; 2 Department of Medicine and Therapeutics, Institute of Digestive Disease, State Key Laboratory of Digestive Disease, LKS Institute of Health Sciences, The Chinese University of Hong Kong, Hong Kong, China

**Keywords:** Crohn’s disease, Biologics, Mucosal healing, Target

## Abstract

Crohn’s disease (CD) is a chronic, progressive, and destructive disease of the gastrointestinal tract. Although its incidence appears to be stable or decreasing in most countries in the North America and Europe, the incidence is rising rapidly in Asian countries. Immunomodulators and biologics are increasingly used to avoid long-term bowel damage and subsequent disability. Therapeutic drug monitoring facilitates optimizing thiopurines and anti-TNFs use. New biologic agents targeting various pathological pathways of CD are blooming in recent years, and the high cost of biologics and expiration of patents for several biologic agents have driven the utility of biosimilars for CD treatment. Here, the literature regarding the efficacy, safety, and withdrawal of the drugs, as well as the evolution of therapeutic targets will be reviewed.

## Introduction

Inflammatory bowel diseases (IBD), including Crohn’s disease (CD) and ulcerative colitis (UC), are chronic idiopathic inflammatory disorders of the gastrointestinal tract, resulting from a combination of genetic predisposition, environmental factors, and inappropriate immune response to the gut microbiota [1]. Since the recognition of the diseases, the incidence of IBD has increased substantially around the world [2]. During the 20th century, IBD prevalence reaches over 0.3% in North America, Oceania, and many countries in Europe [3]. At the turn of the 21st century, while IBD incidence appears to have plateaued in North America and Europe, the incidence of IBD continues to rise among newly industrialised countries in Asia, Africa, and South America [3]. The Asia–Pacific Crohn’s and Colitis Epidemiologic Study (ACCESS), a population-based cohort study of newly diagnosed patients with IBD (2011–2013) from 13 countries in the Asia–Pacific region, has reported that the overall incidence of IBD in Asia is 1.4 cases per 100,000 [4]. Although IBD incidence in Asia is still low at present, with a climbing incidence based on 60% of the global population (https://www.statista.com/statistics/262881/global-population-by-continent/), the absolute number of IBD patients in Asia may catch up with that in the western world over the next decade [5]. In Japan, the prevalence of IBD is also rising based on the National Japan IBD Registry run by the Ministry of Health, Labour and Welfare [6]. Although UC is the predominant type of IBD in Asia, an increase in the incidence ratio of CD to UC over time has been reported [7, 8]. Healthcare costs of CD are almost three times higher than that of UC [9].

## Natural history

CD is a progressive and destructive disease, which may involve the whole gastrointestinal tract [10]. The majority (77–90%) of patients have a chronic intermittent course during 10 years after diagnosis [11, 12]. In a regional inception cohort of 213 Danish CD patients with a follow-up period of at least 7 years, 15% of the patients presented with a worsening change in disease behaviour [13]. A meta-analysis of 198 population-based studies involving 211,563 CD patients showed that corticosteroids, immunomodulators, and biologics are used in 48–60, 32–60, and 6–20% of patients with CD, and the cumulative rates of surgery range from 29 to 49% across different populations [14]. In recent years, immunomodulators and biologics are increasingly used, which is accompanied by a persistent and significant drop of surgery rates for CD [15, 16].

## Evolution of endpoints and treat-to-target

Resolution of symptoms is the primary clinical target for CD treatment. Crohn’s Disease Activity Index (CDAI), which consists of eight variables including the number of liquid stools, the extent of abdominal pain, general well-being, the occurrence of extraintestinal symptoms, the need for antidiarrheal drugs, and the presence of abdominal masses, hematocrit, and body weight, is the standard and most widely used clinical index [17, 18]. Clinical remission is defined as a CDAI score below 150, and clinical response is defined as a decrease of at least 100 points [19], although a lesser cutoff for response with a reduction of at least 70 points was used in some studies [20]. However, the target of symptom control does not appear to significantly alter the natural course of CD [10, 21].

Mucosal healing become the therapeutic goal in clinical practice, as emerging evidence shows that it is associated with a reduced risk of disease relapse [22], hospitalization, and surgery [23, 24]. It has been noted that clinical symptoms as measured by CDAI have poor correlation with mucosal appearance in CD, as half of the patients in clinical remission have endoscopic inflammation, and 20% of patients with mucosal healing have persistent clinical symptoms [25]. The Crohn’s Disease Endoscopic Index of Severity (CDEIS) and Simple Endoscopic Score for Crohn’s Disease (SES-CD) are the most studies endoscopic scoring systems for CD [26]. CDEIS, respectively, scores the presence of deep ulceration, superficial ulceration, length of diseased mucosa, and length of the ulcerated surface in five segments (rectum, sigmoid and left colon, transverse colon, right colon, and ileum). The numbers are summed up and divided by the number of segments evaluated. Additional points are given to stenosis [27]. Endoscopic remission is defined as a CDEIS score of less than 3, and a decrease of over 5 points demonstrates endoscopic response [28]. However, the complexity in calculating the CDEIS score precludes its use in clinical practice [26]. SES-CD, which is scored based on four endoscopic variables (presence and size of ulcers, extent of ulcerated surface, extent of affected surface, and presence and type of narrowing) in the same five segments, provides a practical alternative to the CDEIS [29]. An SES-CD score below 2 is regarded as endoscopic remission [30]. Using relative change in CDEIS and SES-CD scores, a decrease from baseline of at least 50% has been used as the definition for endoscopic response [31]. The Rutgeerts score designed to assess post-operative recurrence, with a score of i2 and above indicating endoscopic recurrence [32]. Although the universal definition for mucosal healing is yet to be determined, the absence of ulceration at ileocolonoscopy has been adopted as the endoscopic endpoint for CD [28, 30].

Given the transmural nature of the disease and inaccessible bowel regions through endoscopy in some cases, imaging modalities (ultrasonography, magnetic resonance imaging, and computed tomography enterography) have been used for assessing disease activity [33]. The most established tool for magnetic resonance imaging is the Magnetic Resonance Index of Activity, which scores wall thickness, relative contrast enhancement, mural edema, and ulcers in different segments of the bowel [34, 35]. While resolution of lesions in imaging examinations has not been broadly accepted as a treatment goal, it has been proposed as a target when endoscopy cannot adequately evaluate inflammation [28].

A patient reported outcome (PRO), which is a measurement derived directly from a patient about any aspect of their health status, without interpretation of their response by a clinician or anyone else [36], has the potential to become a treatment endpoint for CD [37]. Several scales, such as Inflammatory Bowel Disease Questionnaire (IBDQ), Functional Assessment Chronic Illness Therapy-Fatigue (FACIT-F), and Work Productivity Activity Impairment Questionnaire (WPAI), have been used to assess patients’ perspectives towards the disease, although none of them was created according to the United States Food and Drug Administration (FDA) guidance for PRO development [37]. A 2-item interim PRO derived from CDAI has been developed, and resolution of abdominal pain and normalization of bowel habit are proposed to be the primary PRO for CD [28].

To overcome the drawbacks of the traditional progressive, stepwise management of CD for the avoidance of the long-term bowel damage and subsequent disability, a “treat-to-target” strategy has been advocated [38]. The principle of this strategy is to make adjustment of therapy according to regular assessment of disease activity using appropriate treatment targets [39]. A composite target of achieving resolution of abdominal pain and altered bowel habit, and ulceration at ileocolonoscopy as well has been proposed by the International Organization for the Study of Inflammatory Bowel Diseases (IOIBD) [28]. The recommended frequency for assessment is 6–9 months during the active phase [28].

## Conventional medication

### Aminosalicylates

There is consensus that mesalazine has little role for either inducing remission or preventing relapse in CD [19]. Nonetheless, mesalazine and sulfasalazine can be considered in mild colonic CD with superficial lesions [40, 41]. High-dose mesalazine is an option to prevent post-operative recurrence for patients with an isolated ileal resection, and sulfasalazine has beneficial effect on peripheral arthritis associated with CD [42].

### Corticosteroids

Corticosteroids are the mainstay of therapy for inducing remission in CD [40]. Systemic corticosteroids are the first-line treatment to control active disease [19, 41]. A recent network meta-analysis showed that corticosteroids have similar efficacy to high-dose budesonide (above 6 mg/d), but are more effective than high-dose mesalazine for induction of remission [43]. Budesonide is a synthetic steroid that has high topical glucocorticoid activity with a better safety profile compared to conventional corticosteroids, due to its relatively low bioavailability [44]. Therefore, it is recommended in preference to conventional corticosteroids in patients with mildly active localized terminal ileal or ileocecal disease [19].

### Thiopurines

The European Crohn’s and Colitis Organization (ECCO) 2016 guideline advocates thiopurines as the first-line therapy for preventing disease relapse in patients who achieve remission with corticosteroids [41]. It has been confirmed by a meta-analysis that azathioprine (AZA) is superior to placebo for maintenance of remission, with a number needed to treat for an additional beneficial outcome of 9 [45]. The guideline also recommends thiopurines use to prevent post-operative recurrence in patients with higher risk for recurrence [42]. Thiopurine withdrawal is associated with a higher risk for relapse [46]. Randomized controlled trials showed that the relapse rates after stopping AZA ranged from 8 to 25% at 6 months, 16–53% at 12 months, 21–31% at 18 months, and 31% at 24 months, whereas the corresponding proportions were 0, 4–15, 8–12, and 15% in patients who continued on AZA [47–50]. AZA is converted to mercaptopurine (MP) within the thiopurine metabolic pathway [51]. MP is an alternative for AZA-intolerant patients, as MP is tolerated by over two-thirds of patients who are intolerant to AZA [52].

Adverse events of thiopurines lead to drug withdrawal in a significantly higher proportion of patients in randomized clinical trials [45], and the rate reaches up to one-third in some cohorts [53, 54]. The main safety concerns related to thiopurine use are myelotoxicity (4–25%), hepatotoxicity (17%), pancreatitis (4–7%) [55], and an increased risk of various malignancies, including lymphoproliferative disorders, non-melanoma skin cancers (NMSC), myeloid disorders, and urinary tract cancers in a long run [19, 56–59]. The France nationwide prospective observational cohort study—Cancers Et Surrisque Associé aux Maladies inflammatoires intestinales En France (CESAME) has reported an incidence of lymphoproliferative disorder as 0.90 per 1000 [95% confidence interval (CI) 0.50–1.49] patient-years among patients receiving thiopurines, with a multivariate-adjusted hazard ratio (HR) of 5.28 (2.01–13.9, *p* = 0.0007) over those who had never received thiopurines [56]. However, the absolute rate of lymphoma among patients taking thiopurines is low, and the increased risk seems to disappear after discontinuation of the therapy [60]. The CESAME cohort has also revealed that both ongoing thiopurine treatment (HR 5.9; 95% CI 2.1–16.4) and past thiopurine exposure (HR 3.9; 95% CI 1.3–12.1) are risk factors for NMSC [57]. Patients receiving thiopurines have a 2.8 higher risk for urinary tract cancer [59]. Past exposure to thiopurines is associated with a 7-fold increased risk of myeloid disorders [58]. Based on extrapolation from transplant data, it seems that the risks for malignancies begin to significantly accumulate after several years of with thiopurine therapy. It is reasonable to consider thiopurine withdrawal in selected patients after 3–4 years of treatment [61].

Thiopurines are metabolised through three competing pathways via three critical enzymes: hypoxanthine phosphoribosyl transferase (HPRT), thiopurine methyltransferase (TPMT), and xanthine oxidase (XO). MP is methylated to methylmercaptopurine (MMP) by TPMT in one pathway, while in another pathway, it undergoes metabolism to form thioguanine nucleotide (TGN), which is the main therapeutic metabolite of thiopurines [51, 62]. As TPMT deficiency is known to be related to an increased risk of myelotoxicity, to assess TPMT levels prior to thiopurine therapy and adapt the dose accordingly are considered to be useful for the avoidance of serious myelosuppression [40, 51]. Monitoring TGN and MMP levels can guide optimized therapy when an adequate response is not reached: patient education needs to be performed when TGN and MMP are undetectable, which indicates poor drug compliance; patients with low levels of both TGN and MMP require dose increase; allopurinol can be put on to improve the therapeutic efficacy of thiopurine in patients with a low TGN level and a high MMP level, which suggests thiopurine hypermethylation; and patients with TGN within a therapeutic range (235–450 pmol/8 × 10^8^ red blood cells) and a normal level of MMP may be resistant to thiopurine therapy [63]. Recently, the presence of NUDT15 variant has been found to be prevalent amongst Asians and is associated with a higher risk of thiopurine-induced leucopenia [64].

### Methotrexate

Guidelines recommend the use of methotrexate (MTX, 15 mg/week intramuscular) as an alternative to AZA for the maintenance of remission [41]. MTX is recommended in patients with steroid-dependent, steroid-refractory, or AZA-refractory CD, and is also indicated in patients intolerant to other immunomodulating agents [19]. The most common adverse effect of MTX is nausea (in up to 25% of treated patients) [65]. As MTX can include hepatotoxicity and bone-marrow suppression, full blood count and liver function monitoring are warranted [19]. Because of the potential teratogenicity, MTX is contraindicated during pregnancy [19].

### Thalidomide

Refractory CD remains a clinical challenge in more than 20% of patients, despite the advances in biologic therapy [66]. A randomized controlled trial (RCT) of children and adolescents with refractory CD has shown a 46% clinical remission rate at 8 weeks in thalidomide-treated patients and longer duration of remission in responders [67]. Recent studies have found a clinical remission rate of around 50% at 6 months in patients with refractory CD on low-dose thalidomide (50–100 mg/d) [66, 68]. A systematic review, of which 89% are CD patients, has reported an overall rate of sustained clinical remission on thalidomide of 72% at 1 year and an overall rate of complete endoscopic remission of 48% [69]. Lazzerini et al. [70] performed long-term follow-up to patients from two multicenter RCTs, and found mucosal healing and histologic healing achieved in 75 and 53%, respectively, among patients with clinical remission on thalidomide. However, due to the high frequency of major adverse effects, thalidomide should only be considered in short-term use among selected patients [69].

## Anti-tumor necrosis factor agents and drug monitoring

The use of biologics in IBD started with the approval of infliximab, which is an anti-tumor necrosis factor (anti-TNF) agent in CD by the FDA in 1998 [71]. Since then, adalimumab and certolizumab have reached the marketplace for CD treatment [72]. The introduction of anti-TNF has revolutionised the therapeutic strategy of IBD, and its use continues to increase [15]. Well-designed RCTs have demonstrated the efficacy of anti-TNF agents in inducing and maintaining remission in CD [73–84]. A meta-analysis has confirmed the efficacy of all the anti-TNF agents for induction (vs. placebo: relative risks, RR 1.66, 95% CI 1.17–2.36) and maintenance (vs. placebo: RR 1.78, 95% CI 1.51–2.09) of clinical remission. For each anti-TNF agent, infliximab, adalimumab, and certolizumab result in a 3.70-fold (95% CI 0.87–15.80), 2.94-fold (95% CI 1.86–4.66), and 1.22-fold (95% CI 1.00–1.50) higher likelihood of inducing remission, and 1.86-fold (95% CI 1.21–2.86), 2.06-fold (95% CI 1.50–2.82) and 1.62-fold (95% CI 1.30–2.02) higher likelihood of maintaining remission, compared to placebo. There is no evidence of clinical superiority among anti-TNF agents [85]. In another meta-analysis, the pooled rate of mucosal healing is 29% in CD patients treated with anti-TNF (vs. 7% in the placebo arm) at weeks 10–12. Patients on anti-TNF are over three times more likely to achieve mucosal healing than those on placebo. The pooled rates of sustained mucosal healing at week 52–54 are 28 and 1% with anti-TNF or placebo, respectively, with an OR of 19.71 [86]. Anti-TNF agents significantly reduce the risk of hospitalization (OR 0.46, 95% CI 0.36–0.60) and surgery (OR 0.23, 95% CI 0.13–0.42) compared to placebo [87]. A network meta-analysis has shown that anti-TNF is superior to azathioprine in reducing the risk for both of the clinical relapse (RR, 0.11; 95% CI 0.01–0.40) and endoscopic recurrence (RR, 0.04; 95% CI 0.00–0.14) after surgery [88].

ECCO guidelines have swung strongly in favor of early anti-TNF use in CD therapy, especially in patients with high disease activity and features indicating unfavorable prognosis, including young-onset, extensive disease, early need for corticosteroids and perianal disease [19]. Anti-TNF based therapy is also recommended as prophylactic treatment after bowel resection in patients with at least one risk factor for recurrence [42]. Smoking, previous surgery, penetrating disease and perianal involvement are consistently considered as predictors for post-operative recurrence [42, 71].

Up to 40% of patients show no clinical benefit (primary non-response) to anti-TNF therapy [89]. Secondary loss of response refers to the clinical situation, in which a patient has an initial response to anti-TNF, followed by a diminished or less durable response over time. In a recent meta-analysis, the pooled incidence of secondary loss of response with a median follow-up of 1-year is 33%, with an annual risk for loss of response as 21% per patient-year [90]. Immunogenicity failure, defined by the absence of disease improvement in the setting of low anti-TNF levels with high levels of anti-drug antibodies accounts for both of the primary non-response and secondary loss of response in anti-TNF therapy [89]. Combination therapy of an anti-TNF agent with immunomodulator is associated with a reduction in anti-drug antibody formation and better anti-TNF concentrations [91]. The serum levels of anti-TNF significantly correlate with clinical efficacy [92]. The Study of Biologic and Immunomodulator Naive Patient in Crohn’s Disease (SONIC) directly compared the efficacy of infliximab, azathioprine, and the two drugs combined in active CD patients who were naive to both drugs, and found that the combination therapy is superior to infliximab monotherapy in reducing remission (week 26: 56.8 vs. 44.4%; *p* = 0.02) [93]. In a network meta-analysis, the combination of infliximab and azathioprine shows a 98% probability of superiority to infliximab in maintaining remission in CD [94]. In another study, almost half of the IBD patients with anti-adalimumab antibodies and loss of response have sero-reversal of the antibodies, increase in drug trough levels and restoration of clinical response after the addition of a thiopurine or methotrexate [95]. However, the benefits of combination therapy in patients refractory to an immunomodulator before the initiation of anti-TNF is less clear [71]. A systematic review of 11 RCTs with the exclusion of studies involving only subjects naive to anti-TNF and immunomodulator therapy fails to demonstrate a superiority of combination therapy to anti-TNF monotherapy in both reducing and maintaining remission, although subgroup analysis shows the benefit of combination therapy in achieving clinical remission at week 24–30 in patients treated with infliximab who have not previously been exposed to immunomodulators [96].

Therapeutic drug monitoring (TDM) refers to the evaluation of drug concentration and anti-drug antibodies during therapy. As the three possible causes of anti-TNF failure (mechanistic failure, nonimmune-mediated pharmacokinetic failure, and immune-mediated pharmacokinetic failure) present with different levels of serum drug concentration and anti-drug antibodies, TDM can be used to determine the reasons for drug failure and guide the optimization of treatment [97]. The American Gastroenterological Association Institute Guideline suggests reactive TDM in patients with suboptimal disease control on anti-TNF [98]. Secondary loss of response with therapeutic drug trough concentration is regarded as mechanistic failure, in which condition, patients may need to switch to a drug with an alternative mechanism of action, and add an immunomodulator if anti-drug antibodies are detected. The presence of sub-therapeutic drug trough level and undetectable anti-drug antibodies is considered as nonimmune-mediated pharmacokinetic failure, which requires dose escalation. The occurrence of immune-mediated pharmacokinetic failure is indicated when it shows sub-therapeutic drug trough concentration and detectable anti-drug antibodies, in which situation, patients may benefit from switching to a different anti-TNF agent or the addition of an immunomodulator if high level anti-drug antibodies are detected [97]. The suggested therapeutic drug trough concentrations for infliximab, adalimumab, and certolizumab are  ≥ 5,  ≥ 7.5 and ≥ 20 ug/ml, respectively, whereas the uniform thresholds for anti-drug antibody titers remain uncertain [98]. Reactive TDM has been proven more cost-effective than empiric dose escalation for secondary loss of response to anti-TNF treatment [99, 100].

Anti-TNF therapy is relatively safe in CD treatment [101]. Meta-analyses showed no increased risk for serious infection of anti-TNF therapy as compared with placebo in CD [102, 103]. However, the Crohn’s Therapy, Resource, Evaluation, and Assessment Tool (TREAT) Registry, reflecting over 13 years of real-world experience in CD treatment has revealed that compared to other-treatment-only, infliximab is associated with a higher rate of serious infection (2.15/100 vs. 0.86/100 patient-years) [104]. Anti-TNF therapy does not appear to increase the risk for malignancies and mortality [16, 104]. However, its combination with immunomodulator significantly increases the risk of malignancies, including lymphoma and NMSC [105, 106].

Relapse rate after anti-TNF withdrawal is between 30–40% at 1 year, and greater than 50% beyond 2 years [107]. As elevated biological markers (fecal calprotectin and C-reactive protein) and mucosal inflammation associate with higher risk of relapse after anti-TNF discontinuation, patients should achieve biological and endoscopic remission beyond be in clinical remission if anti-TNF withdrawal is considered [61]. Maintenance of immunomodulator treatment after anti-TNF withdrawal reduces the risk for relapse [41, 61]. Retreatment with anti-TNF at relapse is effective with a success rate of 88% [107].

## Other biologics

Vedolizumab, a monoclonal antibody to α_4_β_7_, was approved by the US FDA for the treatment of CD in 2014 [40]. It blocks the α_4_β_7_ integrin on lymphocyte surface that facilitates trafficking of lymphocytes to the gut and the binding of the lymphocytes to gut-specific ligands known as mucosal address in cell adhesion molecule-1 [40]. GEMINI 2 study has evidenced the efficacy of vedolizumab in reducing and maintaining remission in CD. In this study, 14.5% of vedolizumab users achieved clinical remission (vs. 6.8% of those received placebo, *p* = 0.02) at week 6. Clinical remission was maintained over one year in 39 and 36.4% of patients on vedolizumab every 8 weeks and every 4 weeks, respectively (vs. 21.6% of those on placebo, *p* < 0.001 and *p* = 0.004 for the two vedolizumab arms, respectively, vs. placebo) [108]. The utility of vedolizumab as a “rescue” treatment after anti-TNF failed is supported by the GEMINI 3 study [109], and some cohort studies [110–112]. Vedolizumab can induce clinical remission in 20–30% of patients with anti-TNF refractory CD after 6 weeks [109–112]. According to the published data, Vedolizumab is well tolerated with a favorable safety profile [111, 113].

Ustekinumab, an antibody to interleukin-12/23, was approved by the US FDA for the treatment of CD in 2016 [40, 71]. Phase II and phase III trials have shown the superiority of ustekinumab over placebo for the induction and maintenance of clinical remission [114]. In UNITI-1 study, ustekinumab induced clinical remission in 18.5% of patients who failed or intolerant to anti-TNF at week 6 (vs. 8.9% of patients on placebo, – = 0.002) and in 20.9% of these patients at week 8 (vs. 7.3% of patients on placebo, *p* < 0.001) [115]. In UNITI-2 study, ustekinumab led to a higher rate of clinical remission at week 6 (34.9 vs. 17.7% in the placebo arm, *p* < 0.001) and week 8 (40.2 vs. 19.6% in the placebo arm, *p* < 0.001) in patients naive to anti-TNF [115]. IM-UNITI study showed that 2/3 of patients on ustekinumab had sustained clinical remission at week 44 (vs. 45.6% of those on placebo, *p* = 0.007) [115]. CD patients with psoriasis or those who develop anti-TNF induced psoriasis may be the ideal candidates for ustekinumab therapy [19, 114]. Given a favorable safety profile, ustekinumab has potential to become a first-line biologic agent for CD, although much has to be learnt about its long-term safety [114]. Importantly, risk of tuberculosis (TB) is low with this drug, which may be useful in TB endemic regions [116].

## Biosimilars

Healthcare costs for CD have shifted from hospitalization and surgery to expenditures associated with biologic agents, which account for 64% of the total costs [9]. The high cost of biologics and the expiration of patents for several biologics have triggered the development of biosimilar versions of these drugs. The approval of biosimilars follows an expedited process with indication extrapolation [117]. Extrapolated from the results of studied in rheumatoid arthritis, ankylosing spondylitis, or plaque psoriasis, several anti-TNF biosimilar agents have been approved for IBD treatment, and multiple additional agents are currently in development [117, 118]. CT-P13, a biosimilar to infliximab is the first of these agents. Although none of the biosimilars were formally assessed in IBD, the observational “real-world” data so far have shown a comparative efficacy and safety profile of CT-P13 to its reference product infliximab [118, 119]. The NOR-SWITCH study is a randomized controlled trial comparing patients who initiated and continued infliximab to those who initiated infliximab but then switched to CT-P13. The results support switching from infliximab to CT-P13 by showing a non-significant difference in disease worsening at 52 weeks between the two arms. However, it should be noted that more CD patients flared in the CT-P13 arm (36.5 vs. 21.2%), although it did not reach a statistical significance [120].

## Conclusions

A combination of symptom control and mucosal healing has been proposed as the target for CD therapy. Corticosteroids and thiopurines remain as the mainstay of treatment, while anti-TNF agents are increasingly applied earlier in disease course during the recent two decades. Anti-TNF is recommended in patients with high risk for unfavorable prognosis. However, primary non-response or secondary loss of response to anti-TNF therapy occurs in a large proportion of patients, who may benefit from therapeutic drug monitoring. New biologics not only provide hope to patients refractory to anti-TNF, but also has potential to become first-line therapy in selected patients due to beneficial risk profile and long-term efficacy.
